# Hypomethylation at the Regulatory T Cell–Specific Demethylated Region in CD25^hi^ T Cells Is Decoupled from FOXP3 Expression at the Inflamed Site in Childhood Arthritis

**DOI:** 10.4049/jimmunol.1400599

**Published:** 2014-08-04

**Authors:** David Bending, Anne M. Pesenacker, Simona Ursu, Qiong Wu, Hannah Lom, Balathas Thirugnanabalan, Lucy R. Wedderburn

**Affiliations:** * Infection, Inflammation and Rheumatology Section, University College London Institute of Child Health, London WC1N 1EH, United Kingdom; and; † Medical School, Imperial College London, London SW7 2AZ, United Kingdom

## Abstract

The maintenance of FOXP3 expression in CD25^hi^ regulatory T cells (Tregs) is crucial to the control of inflammation and essential for successful Treg transfer therapies. Coexpression of CD25 and FOXP3 in combination with a hypomethylated region within the *FOXP3* gene, called the Treg-specific demethylated region (TSDR), is considered the hallmark of stable Tregs. The TSDR is an epigenetic motif that is important for stable FOXP3 expression and is used as a biomarker to measure Treg lineage commitment. In this study, we report that, unlike in peripheral blood, CD4^+^ T cell expression of CD25 and FOXP3 is frequently dissociated at the inflamed site in patients with juvenile idiopathic arthritis, which led us to question the stability of human Tregs in chronic inflammatory environments. We describe a novel CD4^+^CD127^lo^CD25^hi^ human T cell population that exhibits extensive TSDR and promoter demethylation in the absence of stable FOXP3 expression. This population expresses high levels of CTLA-4 and can suppress T conventional cell proliferation in vitro. These data collectively suggest that this population may represent a chronically activated FOXP3^lo^ Treg population. We show that these cells have defects in IL-2 signaling and reduced expression of a deubiquitinase important for FOXP3 stability. Clinically, the proportions of these cells within the CD25^hi^ T cell subset are increased in patients with the more severe courses of disease. Our study demonstrates, therefore, that hypomethylation at the TSDR can be decoupled from FOXP3 expression in human T cells and that environment-specific breakdown in FOXP3 stability may compromise the resolution of inflammation in juvenile idiopathic arthritis.

## Introduction

Regulatory T cells (Tregs) play central roles in controlling the magnitude of the immune response. Since their identification (1), FOXP3^+^ Tregs have been intensively studied, given their critical roles in regulating immune responses. Before the discovery of FOXP3, high levels of the IL-2 receptor α-chain, CD25 (2), had been relied upon as a marker of T cells with regulatory potential. However, CD25 is not unique to Tregs because T conventional cells (Tcons) upregulate this receptor upon activation. Early studies of FOXP3 (1, 3, 4) demonstrated that the highest CD25-expressing cells were also predominantly FOXP3^hi^, and therefore Tregs were defined by high coexpression of CD25 and FOXP3.

A key paradox often encountered in studies of autoimmune disease, exemplified by childhood arthritis (Juvenile Idiopathic Arthritis [JIA]) (5, 6), is the enrichment of FOXP3^+^ T cells at the inflamed site. This raises the question as to why disease persists despite the enhanced Treg frequency. Several studies have demonstrated that human Tcons upregulate FOXP3 upon activation (7–9), suggesting that, theoretically, any observed CD25^hi^FOXP3^hi^ T cell population could contain activated Tcons (9), as well as Tregs. Work by several groups has shown that epigenetic modifications to the *FOXP3* promoter (10) and intronic enhancer (11, 12) can distinguish Tregs from Tcons. The intronic enhancer region, which has been called the Treg-specific demethylated region (TSDR), is an excellent discriminator between Tregs and promiscuous FOXP3-expressing T cells, and it can be used as a biomarker to quantify Tregs (13). Activated Tcons, TGF-β–induced Tregs, and naive T cells all display methylation of this region. TSDR status can therefore distinguish between a CD25^hi^FOXP3^hi^ activated Tcon and a fully committed CD25^hi^FOXP3^hi^ Treg, and, theoretically, could also be used to identify Tregs that may have recently lost FOXP3 expression.

The terms Treg, FOXP3, and/or CD25^hi^ are sometimes used interchangeably; however, to what extent these markers identify bona fide Tregs at chronically inflamed sites in humans is unknown, and the temporal dynamics of FOXP3 and/or CD25 expression at inflamed sites are poorly understood. Furthermore, functional studies are often hindered in humans because Tregs must be isolated through surrogate cell surface marker expression, for example, CD25 and CD127 (14). We have previously reported that CD25 and FOXP3 expression are frequently dissociated in the synovial fluid (SF) of JIA patients (6), raising questions about the extent and stability of Tregs at the inflamed site. We reveal that hypomethylation at the TSDR is decoupled from stable FOXP3 expression in a subset of CD25^hi^ T cells. This population represents a Treg subset that has very low FOXP3 expression, is proportionally increased in the more severe forms of arthritis, and displays defects in pathways important for Treg homeostasis. Our data suggest that the presence of TSDR hypomethylation is not sufficient in itself to ensure FOXP3 expression.

## Materials and Methods

### Human samples and cells

Blood samples were obtained from 11 healthy adult volunteers and 4 healthy children, all with no known autoimmune or genetic conditions. Forty-one patients with JIA (14 male, 27 female), all fulfilling the International League of Associations for Rheumatology classification criteria (15), were included in this study. Four patients gave only PBMC samples and seven provided both PBMC and SF mononuclear cell (SFMC) samples. Seven patients contributed more than one SF sample. See Supplemental Table I for detailed patient characteristics.

For disease severity analysis, 45 SF samples were analyzed, of which 18 were from patients with the persistent oligoarticular subtype, 16 with extended oligoarticular JIA, and 11 with polyarticular JIA. For clinical severity analysis, the patient’s diagnosis at the time of sampling was identified (including seven patients with serial samples); the polyarticular and extended oligoarticular patient samples were combined as a group because all had more than five joints involved, that is, polyarticular course of disease (more severe, *n* = 27, 70.4% female), and they were compared with the patient samples identifying with the mild remitting persistent oligoarticular JIA subtype (*n* = 18, 61.1% female).

SFMCs and PBMCs were prepared by density gradient centrifugation using LymphoPrep (Axis-Shield) with SF samples undergoing pretreatment with hyaluronidase (10 U/ml; Sigma-Aldrich) (6).

### Flow cytometry and cell sorting

Flow cytometry was performed with the following directly conjugated Abs: CD3 (clone UCHT1) V450/V500 (both BD Biosciences); CD3-PC7 (Beckman Coulter); CD4-FITC (RPA-T4) (eBioscience); CD4 (clone OKT4)-BV711 (BioLegend); CD4 (clone SFCI12T4D11)-PC7 (Beckman Coulter); CD4 (clone S3.5)-QDOT605 (Life Technologies); CD25 (clone M-A251)-PE/allophycocyanin (BD Biosciences); BV421 (BioLegend); CD127 (clone eBioRDR5)-FITC/eFluor 450 (eBioscience); FOXP3 (clone 236A/E7)-allophycocyanin/eFluor 450 (eBioscience); CTLA-4 (clone 14D3)–PE (eBioscience); Ki67 (clone 20Raj1)-eFluor 450 (eBioscience); programmed cell death-1 (PD-1; clone EH12.2H7)–BV605 (BioLegend); p-STAT5 (clone 47/Stat5[pY694])-PE (BD Biosciences); LAP (clone FNLAP)–PE-Cy7 (eBioscience); IL-2 (clone MQ1-17H12)-PE (eBioscience); IL-17A (clone BL168)-BV605 (BioLegend); ubiquitin-specific peptidase 7 (USP7; polyclonal) unconjugated (Bethyl Laboratories) or goat anti-rabbit Alexa Fluor 488 for USP7 detection using standard techniques (see Ref. 16). CTLA-4 staining was performed intracelluarly using the eBioscience FOXP3 staining buffers. Cytokine staining was performed after 2 h of stimulation with 50 ng/ml PMA and 500 ng/ml ionomycin in the presence of 5 μg/ml brefeldin A (all Sigma-Aldrich) before fixation and staining for IL-17A and IL-2 using eBioscience FOXP3 staining buffers. Cell sorting was performed on a FACSAria machine after pre-enrichment of CD4^+^ T cells by immunomagnetic negative selection (StemCell Technologies).

For sorting of cells based on FOXP3 staining, after CD4^+^ T cell enrichment, cells were stained with Live/Dead fixable blue dead cell stain followed by labeling at 4°C with CD4, CD127, and CD25 Abs. Cells were then fixed for 30 min using 1 ml FOXP3 Fix/Perm reagent (eBioscience) before washing and labeling with allophycocyanin-conjugated anti-FOXP3 (clone 236A/E7) for 45 min. Single “live” CD127^lo^ cells were selected for sorting Treg populations according to CD25 and FOXP3 expression status: P1, CD25^lo^FOXP3^hi^; P2, CD25^hi^FOXP3^hi^; P3, CD25^hi^FOXP3^lo^. Tcons were sorted as CD127^hi^CD25^lo^FOXP3^lo^.

### Multispectral imaging flow cytometry

Cells were stained using standard protocols as previously described (see Ref. 16). Multispectral imaging flow cytometry was performed on an Amnis ImageStream^X^ Mark II instrument. Cells were gated on aspect ratio to include only single cells, and the gradient root-mean-square feature was based on DAPI staining to include focused cells. A CD3^+^CD4^+^ gate was then constructed and 1 × 10^4^ images were acquired.

### Detection of phosphorylated STAT5 by flow cytometry

Cryopreserved SFMCs were thawed, washed, and resuspended in plain RPMI 1640 at a density of 5 × 10^6^/ml in FACS tubes (BD Biosciences). Cells were incubated for 15 min at 37°C before stimulation with 100 U/ml recombinant human IL-2 (Roche) for 15 min. Stimulation was stopped by the addition of paraformaldehyde to a final concentration of 1.5% and a further incubation at 37°C for 15 min. Cells were pelleted, permeablized in 90% ice-cold methanol for 15 min on ice, followed by two washes with PBS and one wash with PBS/2.5% FBS. Cells were then stained for 40 min at 4°C with Abs to CD3, CD4, CD25, FOXP3, and p-STAT5.

### DNA extraction

For isolation of DNA from live, unfixed cells, a Qiagen DNeasy blood and tissue kit was used. For isolation of DNA from fixed and permeablized cells, a published protocol was used with minor modifications (17). Sorted cells were incubated with 300 μl lysis buffer (10 mM Tris-HCl, 100 mM NaCl, 50 mM EDTA, 0.5% SDS, 0.1 mg/ml proteinase K [Roche], and 20 μg/ml RNase A [Qiagen], pH 8.0) for 24 h at 60°C on a thermo-shaker. The lysis solution was mixed vigorously with one volume of phenol/chloroform/isoamyl alcohol (25:24:1; Sigma-Aldrich) and the phases were separated by centrifugation at 13,000 rpm for 5 min at room temperature. The aqueous phase was removed and mixed with one volume of chloroform/isoamyl alcohol (24:1), phases were separated by centrifugation at 13,000 rpm for 5 min at room temperature, and the aqueous phase was removed and DNA precipitated by addition of ethanol to a final concentration of 75%. Tubes were incubated at −20°C for at least 4 h, before DNA was pelleted and washed twice with 70% ethanol. DNA pellets were air-dried before dissolving in TE buffer (Sigma-Aldrich), pH 8.0, for at least 24 h at room temperature.

### Bisulfite treatment and methylation analysis

Male patients (patients 5, 9, 24, and 28) were chosen for TSDR analysis, and patients 24 and 28 were chosen for promoter analysis. DNA was bisulfite treated using the EpiTect bisulfite kit (Qiagen). A 336-bp segment containing the TSDR of *FOXP3* was amplified (*FOXP3* TSDR, forward, 5′-TGT TTG GGG GTA GAG GAT TT-3′, reverse, 5′-TAT CAC CCC ACC TAA ACC AA-3′) (11) or the FOXP3 promoter (*FOXP3* promoter, forward, 5′-TGG TGA AGT GGA TTG ATA GAA AAG G-3′, reverse, 5′-TAT AAA AAC CCC TCC CCA CC-3′) (10) by platinum high-fidelity *Taq* (Invitrogen), followed by cloning of PCR products (TOPO TA kit). Clones were sequenced using M13 primers, and sequences were aligned (reverse complementation was accepted) to an imputed TSDR/promoter sequence using Geneious Pro 5.6.6 software (Biomatters). The retention of a C nucleotide at the CpG position identified methylated CpG sites; the presence of a T nucleotide identified unmethylated CpGs. The percentage of clones displaying methylated CpG for each site, as well as total average, was then determined. For TSDR analysis, a mean of 21–24 clones per subset per patient was performed.

### In vitro Treg suppression assays

To assess proliferation of Tcons, Tcons (CD4^+^CD127^hi^CD25^lo^) were labeled in 1 μM CFSE solution as previously described (see Ref. 18). Labeled Tcons were cultured at a constant number of 5 × 10^4^/well either alone (1:0) or at a 1:2, 1:4, and, where cell numbers permitted, 1:8 ratio with either P2-enriched Tregs or P3-enriched Tregs on V-bottom 96-well plates (Costar) precoated with 1 μg/ml anti-CD3 (clone UCHT1, R&D Systems) and 5 μg/ml anti-CD28 (clone CD28.2, BD Biosciences) Abs in culture medium (RPMI 1640 supplemented with 10% FBS and 100 U/ml penicillin/streptomycin) at 37°C and 5% CO_2_ for 4–5 d. Final cell concentration was maintained at 1 × 10^6^/ml. A cytokine multiplex assay was performed as previously described (19).

### In vitro culture of Tcons and TSDR methylation assessment of Tcons

Cells (CD4^+^CD127^hi^CD25^lo^) were isolated from healthy control PBMCs and cultured on anti-CD3 (1 μg/ml)– and anti-CD28 (5 μg/ml)–coated 96-well V-bottom plates at a density of 5 × 10^5^ T cells/ml in the presence of 100 U/ml recombinant human IL-2. On day 7, cells were harvested, stained for CD4, CD25, and FOXP3, plus Live/Dead fixable blue dead cell stain, and CD25^hi^FOXP3^hi^ and CD25^hi^FOXP3^lo^ CD4^+^ T cells were sorted on a FACSAria cell sorter. From the sorted populations, DNA was extracted, bisulfite treated, and the TSDR was amplified, cloned, and sequenced as described above.

### RNA extraction and RT-PCR analysis

RNA was extracted from sorted cells according to the manufacturer’s instructions (TRIzol, Invitrogen) and converted to cDNA as described (see Ref. 18). Primer sequences were: *FOXP3* mRNA, forward, 5′-ACC TGG AAG AAC GCC ATC-3′, reverse, 5′-TGT TCG TCC ATC CTC CTT TC-3′; *USP7* (20), forward, 5′-GCA AGT GCA GAT AGT CGC AGA GGA CC-3′, reverse, 5′-CCA TGG TCT GAG AGA GGC TCT GAA C-3′. Gene expression is displayed relative to *GAPDH* (Qiagen) using the 2^−ΔCt^ method.

### SF Treg culture with IL-2 or proteasome inhibitor MG132

P2 and P3 Tregs were enriched as described above and cultured on 96-well V-bottom plates for 24 h in the presence of medium alone, 2 U/ml recombinant human IL-2, DMSO, or 5 μM proteasome inhibitor MG132 (Sigma-Aldrich).

### Statistical analysis

Statistical analysis was performed on Prism 5 for Macintosh software (GraphPad Software). For clarity, statistical tests and error bar descriptions are included in the figure legends. A *p* value <0.05 was deemed statistically significant.

## Results

### Dissociation of CD25 from FOXP3 expression on CD4^+^ T cells

**FIGURE 1.**
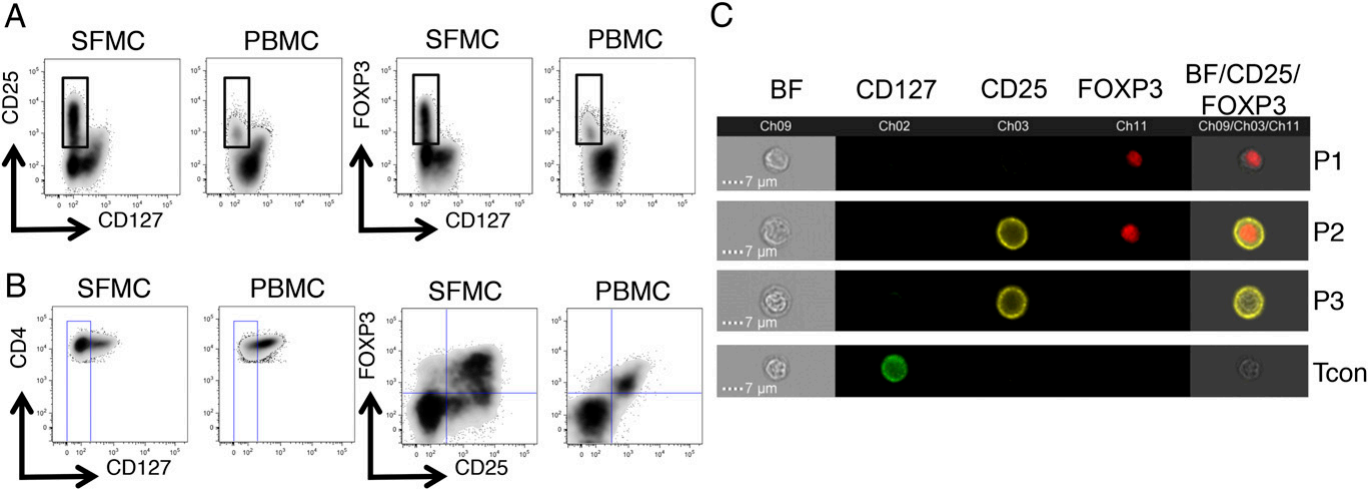
Dissociation of CD25 and FOXP3 expression at the inflamed site. Flow cytometry data, gated on CD3^+^CD4^+^ T cells, of paired SFMC and PBMC samples from a JIA patient displaying (**A**) CD25 versus CD127 expression (*left*) and FOXP3 versus CD127 expression (*right*). Because FOXP3^hi^ and CD25^hi^ cells are almost uniformly CD127^lo^, a CD4^+^CD127^lo^ gate (**B**, *left*) was set to analyze the relationship between FOXP3 and CD25 expression (B, *right*). (**C**) Multispectral imaging flow cytometry was performed on CD4^+^ T cells from SFMCs of a JIA patient. Four representative image panels are displayed illustrating the four populations identifiable by the imaging of CD25 and FOXP3 staining. Brightfield (BF), CD25, and FOXP3 images were merged, with BF intensity set to 40% and CD25 and FOXP3 at 100%.

Analysis of SF and peripheral blood CD4^+^ T cells from JIA patients demonstrated that the CD25^hi^ and FOXP3^hi^ (Fig. 1A) populations are contained within the CD127^lo^ population. Gating on the CD4^+^CD127^lo^ population (Fig. 1B), we observed a marked dissociation of FOXP3 from CD25 in SFMC samples (Fig. 1B) but not in autologous PBMCs. Further analysis of a representative SFMC sample by multispectral imaging flow cytometry validated the flow cytometry data, where the presence of four distinct populations based on the imaging of CD25 and FOXP3 staining are clearly identifiable (Fig. 1C).

### The epigenetic footprint of FOXP3^+^ Tregs is not limited to the CD25^hi^FOXP3^hi^ T cell compartment

Because T cell activation can elicit FOXP3 expression in Tcons (9), we wanted to determine the frequency of bona fide FOXP3^+^ Tregs at the inflamed site. Although there is no single, exclusive Treg marker, the methylation status of the TSDR is a widely used test for distinguishing between activated T cells and bona fide Tregs.

**FIGURE 2.**
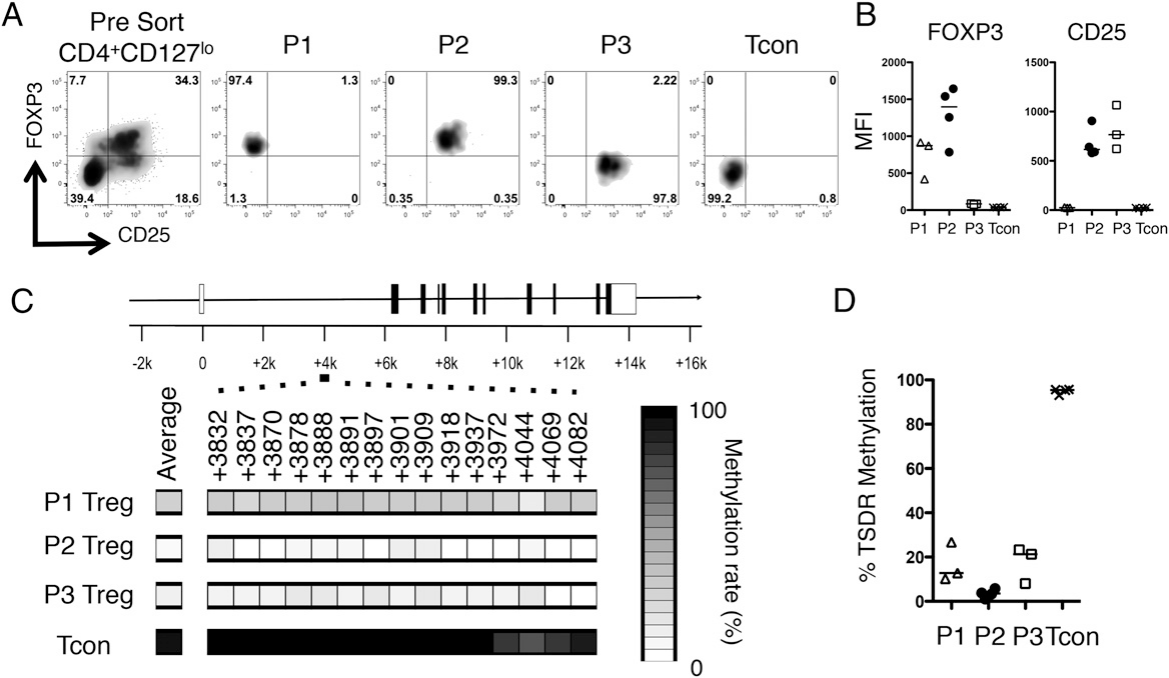
The epigenetic footprint of Tregs is not limited to the CD25^hi^FOXP3^hi^ CD4^+^ T cell compartment. CD4^+^ T cells were isolated from a JIA SFMC sample, stained for CD4, CD25, and CD127, before fixation and staining for FOXP3. Cells were then sorted into four populations based on CD25 and FOXP3 expression. (**A**) Representative sort displaying the presort staining (*far left*, gated on CD4^+^CD127^lo^ T cells) and postsort purities (*right four plots*). The cellular phenotypes were further analyzed for FOXP3 and CD25 protein expression levels. (**B**) Summary plots of MFI data (*n* = 3–4): △, P1 Treg; ●, P2 Treg; □, P3 Treg; **×**, Tcon. (**C**) TSDR methylation rates were determined in the sorted populations from one patient (patient 24) and are presented as heat maps plus region average. (**D**) Summary of the mean TSDR methylation rates in P1–P3 Tregs and Tcons in the four patients studied (patients 5, 9, 24, and 28). P1 Tregs, *n* = 3 (amplification of the TSDR from P1 Tregs in patient 9 was unsuccessful); P2 Tregs, *n* = 4; P3 Tregs, *n* = 3 (patient 5 was excluded from analysis based on a purity of <50%); and Tcons, *n* = 4. Bars represent the median values.

SFMCs were stained for the surface markers CD4, CD25, and CD127 and then fixed, permeablized, and stained for FOXP3 before sorting into four subsets (Fig. 2A), hereafter referred to as follows: P1, CD127^lo^CD25^lo^FOXP3^hi^; P2, CD127^lo^CD25^hi^FOXP3^hi^; P3, CD127^lo^CD25^hi^FOXP3^lo^; and a control population, Tcon, CD127^hi^CD25^lo^FOXP3^lo^, as represented in Fig. 1C. Purities of a representative sort (Fig. 2A) and the summary of FOXP3 and CD25 expression are shown (Fig. 2B, Supplemental Fig. 1A).

DNA was extracted from sorted populations before bisulfite treatment for methylation analysis. Complete methylation was seen in the Tcon population (median, 95.45%). However, interestingly, extensive demethylation was not restricted to the P2 population (Fig. 2C, 2D): the P2 population displayed complete demethylation (median, 3.57%) whereas the P1 population (median, 12.80%) and P3 CD4^+^ T cells (median, 21.3%) also showed extensive demethylation despite the latter two populations returning a small number of clones containing methylated sequences (Supplemental Fig. 1B), indicating that a proportion of these cells may be contaminating activated Tcons. Similar findings regarding ex-FOXP3 T cells have recently been observed in mice (21); however, to our knowledge, this is the first demonstration in humans of a T cell population displaying a decoupling of TSDR demethylation from stable FOXP3 protein expression.

We also investigated the differentially methylated region at the *FOXP3* promoter (10) and similar to P2 Tregs, both P1 and P3 Tregs displayed demethylation at this region (Supplemental Fig. 1C). Interestingly, Tcons also displayed considerable demethylation at several CpG positions, but, as reported by Janson et al. (10), the −77 position was discriminatory with 50% methylation in Tcons compared with <10% in P1–P3 Tregs.

Using an in vitro culture system, we confirmed that activation of Tcons does not promote significant demethylation in T cells regardless of FOXP3 expression levels. In vitro–generated CD25^hi^FOXP3^lo^ and CD25^hi^FOXP3^hi^ cells both showed high levels of methylation (Supplemental Fig. 2).

### P3 T cells display phenotypic characteristics of an activated Treg

**FIGURE 3.**
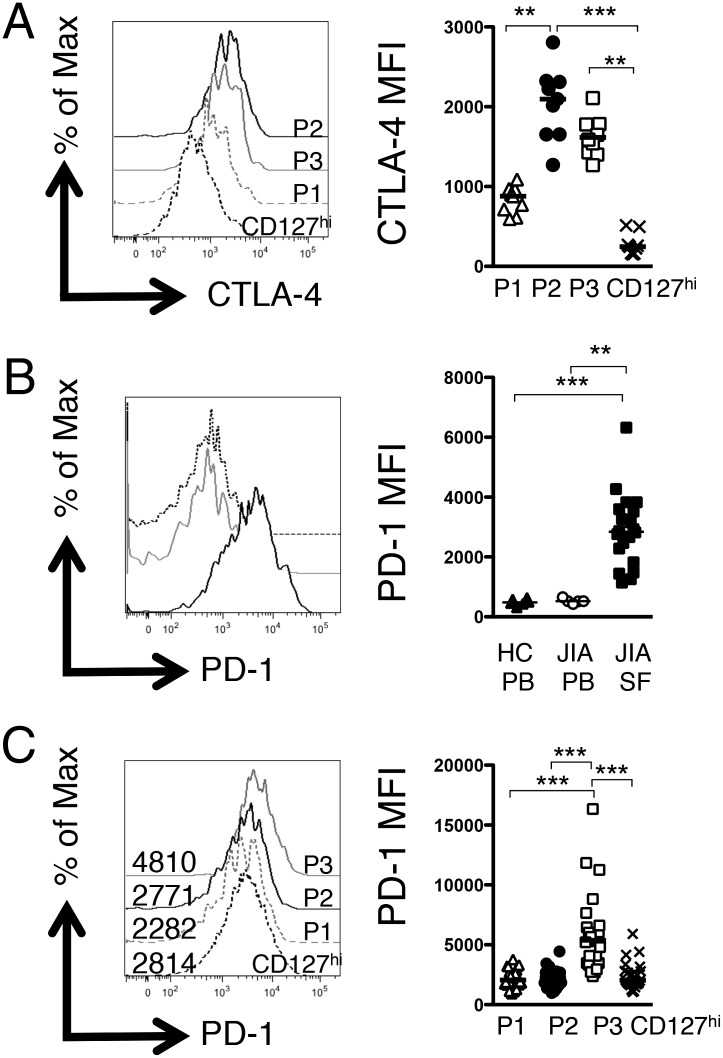
P3 T cells display phenotypic characteristics of an activated FOXP3^lo^ Treg. (**A**) Histogram displaying CTLA-4 levels in the three populations (P1, gray dashed line; P2, black line; P3, gray line) compared with CD127^hi^ T cells (black dashed line) in one representative patient. Summary graph (*right*) displays the MFI of CTLA-4 in the four populations described (*n* = 9). (**B**) Representative histogram overlays (*left*) displaying PD-1 expression levels within the CD4^+^CD127^lo^CD25^hi^ subset in healthy control PBMC (black dashed line), JIA PBMC (gray line), and JIA SFMC (black line) samples; *right*, summary graph of PD-1 MFI levels (adult controls, *n* = 6; JIA PBMCs, *n* = 5; JIA SFMCs, *n* = 20). (**C**) Representative histogram overlays (*left*) showing PD-1 expression (numbers display the MFI) within the four subsets (P1, gray dashed line; P2, black line; P3, gray line; CD127^hi^ T cells, black dashed line); *right*, summary graphs of (*n* = 24) PD-1 protein expression level (MFI). Bars represent median values. Statistical analysis by Friedman test with Dunn’s multiple comparisons (A and C) or Kruskal–Wallis test with Dunn’s multiple comparisons (B). **p < 0.01, ***p < 0.001.

We further examined the phenotype of the three putative Treg populations by assessing intracellular CTLA-4 expression (Fig. 3A). P2 and P3 Tregs exhibited significantly higher levels of total CTLA-4 compared with CD127^hi^ T cells at the inflamed site whereas, interestingly, P1 Tregs exhibited an intermediate level of CTLA-4, which was significantly lower than that of P2 Tregs.

Based on high CD25 and CTLA-4 expression, as well as a demethylated TSDR but low FOXP3 expression, we hypothesized that P3 Tregs may represent a chronically activated FOXP3^lo^ Treg phenotype. The marker PD-1 can be expressed on chronically stimulated CD8 T cells and is associated with T cell exhaustion in HIV-infected individuals (22). We therefore determined the expression levels of PD-1. Whereas PD-1 expression on all SF CD127^lo^CD25^hi^ cells was significantly higher than on controls (Fig. 3B), we observed significantly higher levels of PD-1 on the P3 Tregs compared with P1, P2, or CD127^hi^ T cells (Fig. 3C).

### P3 Tregs display a modest functional inferiority compared with P2 Tregs

**FIGURE 4.**
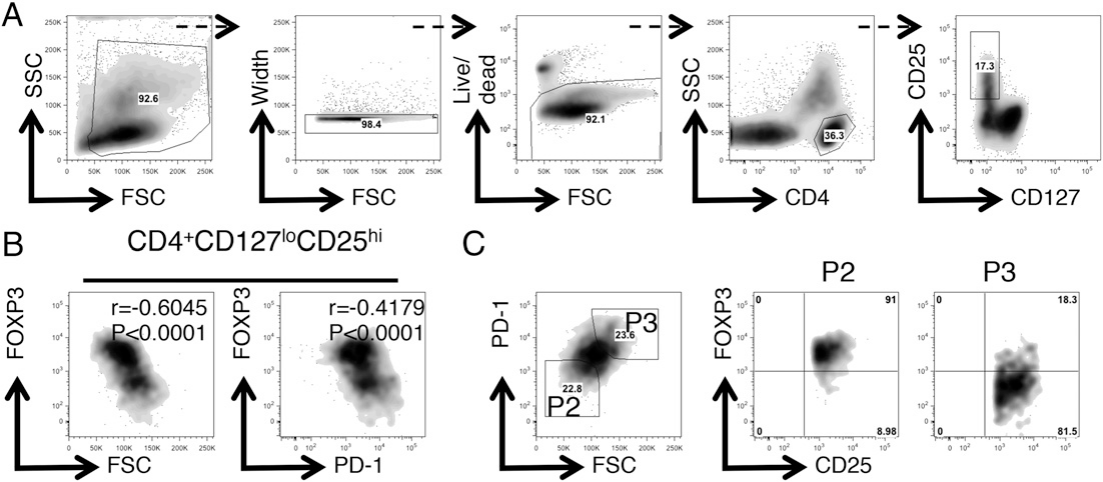
PD-1 expression and FSC characteristics partition the CD25^hi^CD4^+^ SF T cell compartment into P2 and P3 Treg subsets. Displayed is an SFMC sample stained for CD4, CD127, CD25, FOXP3, and PD-1 and analyzed by flow cytometry. (**A**) Boolean gating strategy used to obtain the CD127^lo^CD25^hi^CD4^+^ T cell population. (**B**) Flow cytometry plots gated on CD4^+^CD127^lo^CD25^hi^ SF T cells displaying FOXP3 versus FSC (*left*) and FOXP3 versus PD-1 expression (*right*). Intrasample correlations were analyzed by the Spearman rank correlation analysis; *n* = 1856 data pairs. ****p < 0.0001. (**C**) Flow cytometry data gated on CD4^+^CD127^lo^CD25^hi^ SF T cells (*left*) displaying PD-1 versus FSC expression. Flow cytometry plots (*right*) display CD25 versus FOXP3 expression on the cells in the gates P2 and P3.

**FIGURE 5.**
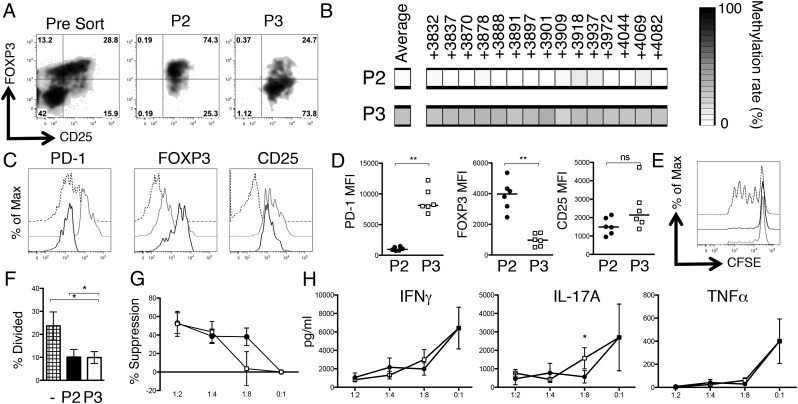
P3 Tregs display a modest functional deficit. (**A**) Flow cytometry plots displaying CD25 versus FOXP3 expression in unsorted sample from patient 21 (*left*), P2-enriched Tregs, CD127^lo^CD25^hi^FSC^lo^PD-1^int^ (*middle*), and P3-enriched Tregs, CD127^lo^CD25^hi^FSC^hi^PD-1^hi^ (*right*). (**B**) TSDR methylation rates in sorted Treg populations from patient 21. (**C**) Representative histogram overlays (*top*) displaying expression levels of PD-1, FOXP3, and CD25 in the sorted Treg populations compared with autologous SF Tcons (P2, black line; P3, gray line; CD127^hi^ T cells, black dashed line). (**D**) Summary graphs show MFI data for PD-1, FOXP3, and CD25 in the sorted samples (*n* = 6). CFSE-labeled SF Tcons were cultured alone (0:1) or at a 1:2, 1:4, or 1:8 ratio with either P2 Tregs or P3 Tregs on anti–CD3/CD28-coated plates for 4–5 d. (**E**) Histogram overlay displaying Tcon CFSE dilution in the presence of P2 Tregs (black line) and P3 Tregs (gray line) at a 1:2 ratio. Tcon alone division is displayed in black dashed line. (**F**) Summary data demonstrating percentage of divided Tcons in the presence of P2 (filled bar) or P3 (open bar) Tregs (*n* = 6) or no Tregs (hatched bar). (**G**) Percentage suppression of SF Tcons at 1:2 (*n* = 6), 1:4 (*n* = 6), and 1:8 (*n* = 5) Treg/Tcon ratios. (**H**) Levels of IFN-γ, IL-17A, and TNF-α in coculture supernatants. Tcons plus P2 Tregs (●) and Tcons plus P3 Tregs (□) are shown. Bars represent the median value in (D) and means ± SEM in (F)–(H). Statistical analysis was performed by a Mann–Whitney *U* test (D), repeated measures ANOVA with a Bonferroni multiple comparisons test (F), or a paired *t* test (H). *p < 0.05, **p < 0.01.

To examine the regulatory potential of the FOXP3^lo^ cells observed at the site of inflammation, we required a way to separate viable cells. We hypothesized that higher levels of PD-1 could facilitate the separation of the CD25^hi^ population into FOXP3^hi^ and FOXP3^lo^ populations in the patient samples displaying the biggest differences in PD-1 median fluorescence intensity (MFI) between the two Treg populations. Additionally, during postsort purity analyses we noted that P3 Tregs had higher forward light scatter (FSC) characteristics than did P2 Tregs. We therefore developed a sort strategy to separate the CD25^hi^ population into FOXP3^hi^ and FOXP3^lo^ populations (Figs. 4, 5A, Supplemental Fig. 3A), enabling an estimation of their functional capacities. To validate that this sort strategy did enrich for TSDR demethylated T cells, TSDR analysis was performed on cells from patient 21 (Fig. 5B). Postsort expression of CD25, FOXP3, and PD-1 were also analyzed. Significant differences were found in PD-1 MFI (>8-fold, as expected) and FOXP3 MFI (>4-fold, as predicted), but not in CD25 expression (Fig. 5C, 5D).

P2- and P3-enriched Tregs were tested for their ability to inhibit Tcon proliferation (Fig. 5E–G) and cytokine secretion (Fig. 5H) at a range of Treg/Tcon ratios. Both subsets significantly suppressed Tcon proliferation at a 1:2 ratio of Tregs to Tcons (Fig. 5E, 5F). Titration of Tregs suggested that the suppressive capacity of P3-enriched Tregs was lower than that of P2 at the lower Treg/Tcon ratio of 1:8; however, the differences between P2 and P3 did not reach statistical significance (*p* = 0.0787 [paired *t* test] at a 1:8 ratio, Fig. 5G).

Supernatants from these cultures were analyzed for levels of IFN-γ, IL-17A, and TNF-α by cytokine multiplex assay. Both P2- and P3-enriched Tregs suppressed IFN-γ and TNF-α production to a similar degree; however, there was a significant difference in the suppression of IL-17A at the 1:8 Treg/Tcon ratio (*p* = 0.0215) between P2 and P3 Tregs (Fig. 5H). Given the recent reports that FOXP3 protein downregulation can lead to a de-repression of cytokine genes, such as IL-2 (20), we investigated cytokine production by P1–P3 Tregs (Supplemental Fig. 3B, 3C). We found no difference in latency-associated peptide expression, but IL-2– and IL-17A–producing cells were slightly increased in frequency in P3 Tregs when compared with P2, which in part could have contributed to the differences in the suppression of IL-17A secretion.

Of note, P2-enriched Tregs maintained their FOXP3^hi^ phenotype during the in vitro culture, and interestingly P3-enriched Treg FOXP3 protein levels remained significantly lower (FOXP3 MFI at 1:2 ratio of 2433.5 versus 799.8 for P2 versus P3 Tregs, *p* < 0.01).

### Defective IL-2 signaling in P3 Tregs

Our results suggested that high FOXP3 expression is not essential for the capacity of T cells to act as regulators, but that it may be important for optimal suppression. We next explored possible mechanisms to explain the loss of FOXP3 expression in P3 Tregs.

**FIGURE 6.**
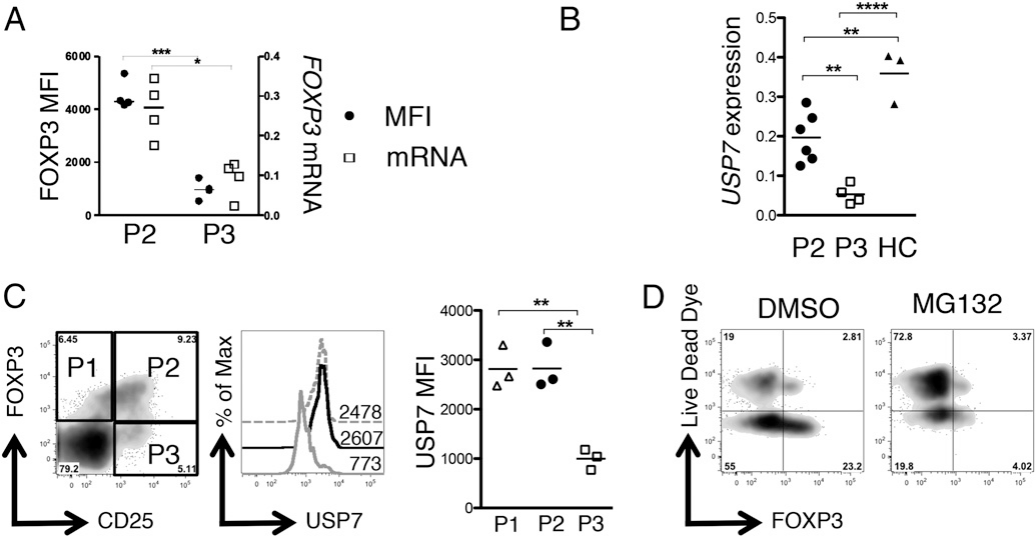
Decreased *FOXP3* mRNA and expression of *USP7* in P3 Tregs. RNA was extracted from P2- and P3-enriched Treg populations and converted to cDNA. (**A**) FOXP3 protein MFIs (●) and mRNA levels of *FOXP3* (relative to *GAPDH*) (□) in P2- and P3-sorted subsets (*n* = 4). (**B**) Relative levels of *USP7* mRNA were analyzed by RT-PCR in P2-enriched Tregs (*n* = 6), P3-enriched Tregs (*n* = 4), or healthy control Tregs (*n* = 3). (**C**) *Left*, Flow cytometry plot showing CD25 versus FOXP3 staining gated on CD4^+^ T cells in a SF sample, with gates for P1, P2, and P3 Tregs. *Center*, Histogram overlay showing USP7 staining in P1 (gray dashed line), P2 (black line), or P3 Tregs (gray line). Numbers represent MFIs (*n* = 3). *Right*, Summary data showing MFI. (**D**) To examine FOXP3 degradation, P3-enriched SF Tregs were cultured for 24 h in the presence/absence of 5μM proteasome inhibitor MG132. Expression of FOXP3 versus Live/Dead dye was analyzed by flow cytometry. Statistical analysis performed by a Mann–Whitney *U* Test (A) or a one-way ANOVA with a Bonferroni multiple comparisons test (B and C). *p < 0.05, **p < 0.01, ***p < 0.001, ****p < 0.0001.

Two recent studies have highlighted a previously unappreciated role for the ubiquitin pathway in regulating FOXP3 expression (20, 23). We hypothesized that if posttranslational mechanisms were dominant in regulating FOXP3 protein levels, then mRNA levels for *FOXP3* should be comparable between P2 and P3 Tregs; alternatively, if transcriptional regulation were the primary mechanism, then *FOXP3* transcript levels should be significantly lower in P3 versus P2 Tregs. Analysis of expression levels of *FOXP3* in P2- and P3-enriched Treg populations showed that FOXP3 protein levels and relative *FOXP3* mRNA expression were both significantly lower in P3-enriched Tregs (Fig. 6A). This finding, however, did not exclude the possibility that posttranslational mechanisms may also play a role in regulating FOXP3. The deubiquitinase USP7 is upregulated in Tregs and prolongs the half-life of FOXP3 protein by reducing its ubiquitin-mediated degradation rate (20). We measured the levels of *USP7* mRNA by RT-PCR (Fig. 6B) and flow cytometry (Fig. 6C) and found that P3 Tregs exhibited a significantly lower level of *USP7* compared with healthy control or P2 Tregs. To assess whether protein degradation was an important factor in generating the P3 phenotype, we cultured P3-enriched Tregs with the proteasome inhibitor MG132: however, we observed no increase in FOXP3 staining after culture in MG132, which may be in part due to the toxicity of MG132 to SF Tregs (Fig. 6D). These findings collectively suggest that maintenance of USP7 expression may be important for optimal FOXP3 expression at the inflamed site, but that proteasomal degradation alone may not explain the FOXP3^lo^ phenotype.

**FIGURE 7.**
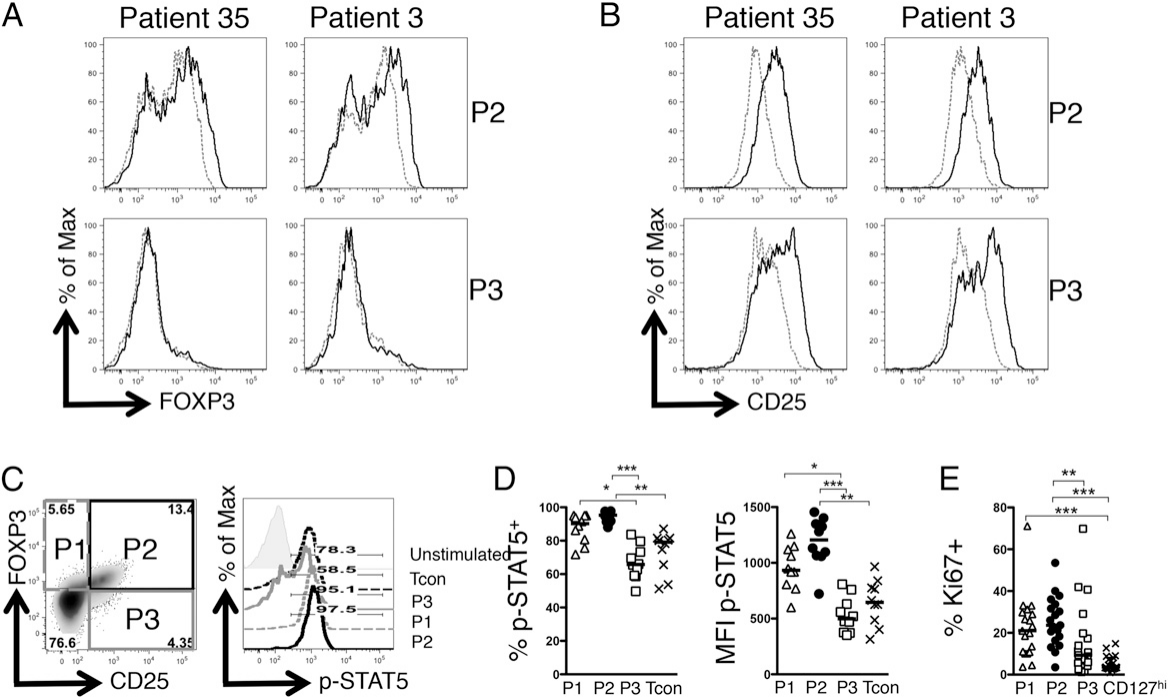
Decreased IL-2 responsiveness in P3 Tregs. (**A**) P2- and P3-enriched SF Tregs were isolated as previously described and cultured for 24 h with or without 2 U/ml IL-2. Histograms of Tregs 24 h after culture displaying FOXP3 (A) or CD25 (**B**) expression are shown. To assess IL-2 sensitivity, total SFMCs from JIA patients were stimulated with 100 U/ml recombinant human IL-2 for 15 min. (**C**) Flow cytometry plot (*left*) gated on CD3^+^CD4^+^ T cells, showing CD25 versus FOXP3 expression. Histogram (*right*) displaying p-STAT5 levels in stimulated P1 (gray dashed line), P2 (black line), and P3 (gray line), or CD25^lo^FOXP3^lo^ T cells (black dashed line); unstimulated T cell p-STAT5 levels are represented by the filled histogram. (**D**) Summary graphs of percentage p-STAT5^+^ (*left*) or MFI of p-STAT5 protein (*right*) in the four different T cell populations (*n* = 10; data are from five independent experiments). (**E**) Graph of percentage Ki67^+^ cells in the three Treg subsets compared with CD127^hi^ T cells (*n* = 19). Bars display median values. Statistical analysis was performed by Friedman test with Dunn’s multiple comparisons test (D and E). *p < 0.05, **p < 0.01, ***p < 0.001.

We next considered the transcriptional regulation of *FOXP3* expression. The importance of IL-2 in maintaining FOXP3 expression is well established (24). Because both P2 and P3 Tregs expressed high levels of CD25, we hypothesized that both should be able to respond to IL-2. We determined whether IL-2 exposure would be sufficient to recover FOXP3 expression in P3 Tregs. Both P2- and P3-enriched Tregs were sorted from SF and cultured for 24 h with or without IL-2, and expression of CD25 and FOXP3 was analyzed (Fig. 7A, 7B). P2-enriched Tregs displayed a modest increase in FOXP3 expression and a positive shift in CD25 expression in response to IL-2; however, P3-enriched Tregs showed no increase in FOXP3 levels and a nonuniform increase in CD25 expression. The observation of this bimodal response to IL-2, as well as a previous report that Tregs in patients with autoimmunity can display defective IL-2 signaling (25), led us to hypothesize that a proportion of P3 Tregs may have a defect in signaling in response to IL-2. We therefore examined IL-2–induced STAT5 phosphorylation in SF T cells (Fig. 7C, 7D).

SFMCs from JIA patients were cultured with IL-2 before analysis (Fig. 7C, 7D). P2 and P1 cells exhibited robust responses to IL-2 with >90% of cells responding, as indicated by phosphorylation of STAT5 (Fig. 7C). In contrast, P3 cells exhibited a median 65.8% response, which was significantly lower than the P1 or P2 Tregs, and even had a trend to lower STAT5 phosphorylation levels than did Tcons (median, 79.15%; Fig. 7D, *left*). Levels of p-STAT5 protein quantified by MFI followed the same significant patterns (Fig. 7D, *right*).

Given the critical role for IL-2 in promoting Treg homeostasis, we hypothesized that P3 Tregs would display diminished turnover in vivo. Both P1 and P2 Treg populations had a significantly higher rate of turnover compared with CD127^hi^ T cells (median frequencies: P1, 21.00%; P2, 23.60%; CD127^hi^, 3.65%) whereas P3 exhibited intermediate turnover (median frequency, 9.28%), which was not significantly different from Tcons (Fig. 7E), suggesting that defects in IL-2 sensitivity may result in a failure to maintain FOXP3 expression and reduced proliferative potential for P3 Tregs.

### Increased proportions of P3 Tregs in the more severe forms of childhood arthritis

**FIGURE 8.**
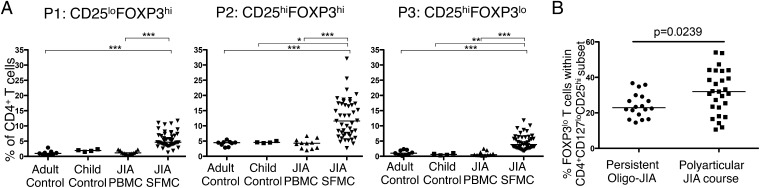
Increased proportions of FOXP3^lo^ Tregs in the more severe forms of childhood arthritis. (**A**) Using the gating strategy determined in Fig. 1, the frequency of P1, P2, and P3 Tregs within the CD4^+^CD127^lo^ T cell compartment was determined in adult control PBMCs (*n* = 9), child control PBMCs (*n* = 4), JIA patient PBMCs (*n* = 11), and JIA patient SFMCs (*n* = 45). Bars represent median values. (**B**) The proportion of FOXP3^lo^ cells within the CD4^+^CD127^lo^CD25^hi^ T cell compartment was analyzed according to disease severity at the time of sample: persistent *O*-JIA, *n* = 18; polyarticular JIA course, *n* = 27. Statistical analysis by Kruskal–Wallis test with a Dunn’s multiple comparisons test (A) or Mann–Whitney *U* test (B). *p < 0.05, **p < 0.01, ***p < 0.001.

To understand the clinical significance of P3 Tregs, we next analyzed the frequency of the Treg populations in control subjects as well as peripheral blood and SF of patients with JIA. All three populations were significantly increased in frequency in the inflammatory compartment compared with controls or JIA PBMCa, although P1 and P3 SF Treg frequencies displayed non-Gaussian distributions (Fig. 8A).

We next investigated whether the proportions of FOXP3^lo^ Tregs within the CD25^hi^ T cell compartment had any relationship to disease severity in JIA. It has been previously demonstrated that the milder form of oligoarticular JIA (*O*-JIA), called persistent *O*-JIA, is associated with an increased frequency and number of Tregs in the inflamed compartment (5, 6) compared with the more severe forms of disease; however, these studies did not stratify according to FOXP3 high or low expression. We observed a significantly increased proportion of FOXP3^lo^ T cells within the CD127^lo^CD25^hi^ T cell compartment in patients with more severe disease (median, 31.97 versus 22.92%, *p* = 0.0239) when the samples were split according to the disease severity at the time of sampling (severe, polyarticular disease course; mild, persistent *O*-JIA; Fig. 8B). Although the frequency of patients on methotrexate at the time of sampling was increased in the polyarticular course group compared with the persistent JIA group, this did not reach statistical significance (55.6 versus 27.8%, *p* = 0.077, Fischer exact test). This result suggests that the presence of FOXP3^lo^ Tregs within the CD25^hi^ Treg compartment may have consequences for disease pathogenesis.

## Discussion

The stability of the FOXP3^+^ Treg lineage has been a controversial issue for some time. We have demonstrated that high levels of CD25 and FOXP3 coexpression successfully identify committed and functional Tregs, even at the site of inflammation; however, within the CD25^hi^ population there is a notable decoupling of TSDR demethylation from FOXP3 expression in a subset of cells. Our data strongly support the case that this CD25^hi^FOXP3^lo^ TSDR demethylated population is a FOXP3^lo^ Treg population that we speculate may have downregulated FOXP3 in the inflammatory environment.

Another striking feature of this study is the considerable heterogeneity in Treg subsets that is present at the inflamed site in humans. Whereas PBMCs show more discrete clusters, it is clear that the proportions of P1–P3 Tregs vary considerably between individuals, as well as each subset displaying its own heterogeneity within (notably P3 Tregs). A possible explanation for this is that the cells may arise from differing local environments within the joint or may be in differing stages of activation.

One unifying feature, however, of P1–P3 Tregs is that they are predominantly CD127^lo^, which is interesting given the observations that some Tregs can upregulate CD127 upon activation in mice (26). In our hands, a common theme is that the joint environment promotes a downregulation of CD127 and a concomitant upregulation of CD25. Indeed, factors that govern the expression at the inflamed site of these receptors warrants further investigation, given the importance of common γ-chain cytokines to T cell survival.

Our data demonstrate that the P1 and P3 phenotypes are largely confined to the inflamed envrionment. One limitation when working with human SF is the absence of an appropriate biological control. The joints of healthy individuals are largely acellular, and ethical implications preclude the removal of SF from healthy controls. It will be interesting to compare across other diseases, including both autoimmune and nonautoimmune inflammatory disorders, as to whether the P3 Treg is a consequence of chronic inflammation, or is particular to autoimmunity. Furthermore, although this study has focused on P3 Tregs, it is clear that CD25^lo^ Tregs (P1) are also of interest and worth investigating. Such cells have been reported in the blood of lupus patients (27, 28), and therefore further work is required to understand the co-regulation of CD25 and FOXP3 expression on Tregs, and how this may become dysregulated. We would hypothesize from our data that P1 Tregs represent a P2 Treg population that has downregulated CD25 expression.

It is increasingly clear that the maintenance of high FOXP3 expression in Tregs is complex and dependent on multiple factors. Undoubtedly epigenetic modifications are important players in determining the stable commitment of Tregs; in this case, the TSDR status (11, 12, 29) has been widely used to define stable FOXP3^+^ Tregs. Although it is known that epigenetic programming of Tregs is essential for their development (29), our data show that TSDR demethylation alone is not sufficient in itself to maintain high FOXP3 protein expression, and other factors affecting both transcription and protein stability are defective in a subpopulation of CD25^hi^ Tregs.

The observed loss of FOXP3 protein expression in Tregs does not necessarily support the conclusion that the entire Treg program is intrinsically “unstable” at the inflamed site, but reflects the complexity of the regulation of FOXP3 expression, and also that a Treg is more than just a FOXP3-expressing cell (29). P3 Tregs display many characteristics of Tregs, and as such we do not think that they represent an ex-Treg population, but speculate that they may represent a FOXP3^+^ Treg that has downregulated FOXP3 but retains many features of Tregs, including the ability to suppress immune responses. Although in this study we cannot prove that P3 Tregs have previously expressed FOXP3, the concept of an ex-FOXP3 T cell has been reported in mice (30), and such cells can re-express FOXP3 following culture in vitro (21). The authors suggested that epigenetic modifications to the *FOXP3* locus might serve to maintain Treg lineage commitment by imprinting memory for FOXP3 (and thus Treg commitment) regardless of ongoing FOXP3 expression. Our findings are consistent with these observations, because TSDR and promoter demethylated human FOXP3^lo^ Tregs still displayed suppression in vitro. Furthermore, fate mapping in mice has shown that, at the very least, a clear subset of the Treg lineage maintains stable *FOXP3* transcription, even under inflammatory conditions (31). Most fate-mapping studies, however, have deployed genetic constructs that use transcriptional readout for *FOXP3* expression, and they cannot be solely relied upon to predict FOXP3 protein stability. Recent insight into the posttranslational control of FOXP3 protein may explain reported differences in studies investigating Treg FOXP3 expression stability, as several groups have documented that inflammatory insults, such as LPS challenge, IL-6, or CCL3, can trigger the ubiquitination and degradation of FOXP3 protein, which can downmodulate Treg function without affecting *FOXP3* gene expression (20, 23, 32). Analysis of these Treg populations by transcriptional methods alone would have failed to identify this downregulation of FOXP3 protein.

The in vivo consequences of human Treg FOXP3 downregulation are difficult to gauge since the studies of human Treg suppression are largely limited to the in vitro setting. Our data suggest that although P3 Tregs are suppressive in an ex vivo culture system, their performance in vivo may be compromised, as increased proportions of FOXP3^lo^ Tregs are found in patients with more severe disease course of JIA. It is clear from the literature that many immune cell populations displaying robust in vitro suppressive capacity are unable to regulate in vivo, where factors in addition to FOXP3 stability, such as cellular migration, turnover, Ag specificity, and survival, are likely to be important. To this end, there is a great need to develop more sophisticated assays to investigate human Treg function.

We observed that P3 Tregs displayed reduced sensitivity to IL-2 signals, and this in part may explain why IL-2 stimulation was insufficient to recover FOXP3 protein levels in these cells despite the well-known report that IL-2 is critical for maintaining Treg FOXP3 expression (24). Defects in IL-2 signaling have been observed in patients with autoimmunity and have been attributed to increased expression of the phosphatase PTPN2 (25). In our hands, experiments involving the phosphatase inhibitor sodium vanadate did not alter IL-2 signaling in P3 Tregs (D. Bending, unpublished observation), and the mechanisms behind this disruption in STAT5 responsiveness is of interest given that IL-2 is important not only in supporting FOXP3 expression but also for Treg proliferation and survival. Although P2 Treg turnover at the inflamed site is significantly higher than that of Tcons, as previously reported (33), it is tempting to speculate that defects in IL-2 signaling may also render P3 Tregs hypoproliferative, and the inability of FOXP3^lo^ Tregs to proliferate and upregulate FOXP3 in vivo may affect their capacity to adequately respond to and control autoimmune responses. This has important ramifications for strategies deploying adoptive Treg therapies to treat inflammatory disorders, because transferred TSDR-demethylated Tregs may lose FOXP3 under conditions of stress or inflammation. It will be essential to further clarify the precise mechanisms by which Tregs can lose FOXP3 expression to optimize therapeutic strategies.

Past evidence in JIA suggests that the more severe form of oligoarthritis (extended *O*-JIA) is associated with a reduced frequency of Tregs compared with the milder form of disease (5, 6). Clinical subtype analysis performed on all the SF samples in our study revealed a significant correlation between the frequency of FOXP3^lo^ Tregs within the CD25^hi^ T cell population and disease course. We therefore hypothesize that the local environment may play a critical role in regulating FOXP3 expression at inflamed sites, and that some Tregs may downregulate FOXP3. We suggest that the P3 Treg phenotype is analogous to a recently described ex-FOXP3 Treg subset identified in experimental autoimmune encephalomyelitis (30). It would be in keeping with murine experiments that downregulation of FOXP3 in Tregs results in reduced regulation and worsened disease, as evidenced experimentally in models of type 1 diabetes, where Treg ablation leads to almost immediate onset of disease (34). Gleaning a greater knowledge regarding the nature of, and mechanisms involved in, cells that lose FOXP3 in vivo will aid in our understanding of how Tregs may fail to function adequately in disease settings.

We have described in the present study that stable FOXP3 expression is decoupled from TSDR demethylation in a population of joint-infiltrating Tregs in human arthritis, and that the proportional representation of such cells within the Treg subset correlates with disease severity. Given the growing evidence that downregulation or loss of FOXP3 in Treg populations is detrimental to the control of chronic inflammation (20, 23, 34), future work should try to clarify the mechanisms that occur in vivo to regulate CD25 and FOXP3 expression. A detailed understanding of these processes may yield new and unexpected therapeutic avenues to exploit for the treatment of debilitating autoimmune diseases, such as JIA.

## Supplementary Material

Data Supplement
