# Supplementary material for: Hypomethylation at the Regulatory T Cell–Specific Demethylated Region in CD25hi T Cells Is Decoupled from FOXP3 Expression at the Inflamed Site in Childhood Arthritis
Source: J Immunol. 2014 Aug 4;193(6):2699–708. doi: 10.4049/jimmunol.1400599 (PMC4157061; doi:10.4049/jimmunol.1400599)
Supplement: Data Supplement [file supp_193_6_2699__index.html]

Hypomethylation at the Regulatory T Cell–Specific Demethylated Region in CD25hi T Cells Is Decoupled from FOXP3 Expression at the Inflamed Site in Childhood Arthritis — Data Supplement 

# Hypomethylation at the Regulatory T Cell–Specific Demethylated Region in CD25hi T Cells Is Decoupled from FOXP3 Expression at the Inflamed Site in Childhood Arthritis

## Data Supplement

**Files in this Data Supplement:**

- Supplemental Material 1 (PDF)
