# Supplementary material for: Hypomethylation at the Regulatory T Cell–Specific Demethylated Region in CD25hi T Cells Is Decoupled from FOXP3 Expression at the Inflamed Site in Childhood Arthritis
Source: J Immunol. 2014 Aug 4;193(6):2699–708. doi: 10.4049/jimmunol.1400599 (PMC4157061; doi:10.4049/jimmunol.1400599)
Supplement: Data Supplement [file 1400599_JI_1400599_Supplemental_Material_1.pdf]

Supplementary Table 1

Patient ID	SFMC	PBMC	Gender	Serial Samples	JIA subtype	Figures
**1**	No	Yes	M	No	Extended Oligo	3B, 8A
**2**	Yes	No	F	No	Polyarticular	3B, 3C, 7D, 7E, 8A, 8B
**3**	Yes	Yes	M	Yes	Extended Oligo	3B, 3C, 7A, 7B, 7D, 7E, 8A, 8B, S3B
**4**	Yes	No	F	No	Extended Oligo	3A, 3B, 3C, 6B, 8A, 8B
**5**	Yes	No	M	Yes	Persistent Oligo	2B, 2D, 6C, 8A, 8B, S1A
**6**	Yes	Yes	F	No	Extended Oligo	3B, 3C, 7D, 7E, 8A, 8B
**7**	Yes	No	F	No	Extended Oligo	3A, 3B, 3C, 7D, 7F, 8A, 8B
**8**	Yes	No	F	No	Extended Oligo	3B, 3C, 8A, 8B
**9**	Yes	No	M	No	Extended Oligo	2B, 2D, 8A, 8B, S1A
**10**	Yes	No	F	No	Extended Oligo	3A, 3B, 3C, 7D, 7E, 8A, 8B
**11**	Yes	No	F	No	Persistent Oligo	3A, 3B, 3C, 7D, 7E, 8A, 8B
**12**	No	Yes	M	No	Persistent Oligo	8A
**13**	No	Yes	M	No	Persistent Oligo	8A
**14**	Yes	Yes	F	No	Persistent Oligo	3B, 3C, 8A, 8B
**15**	Yes	Yes	F	yes	Extended Oligo	3B, 3C, 7D, 7E, 8A, 8B
**16**	No	Yes	F	No	Persistent Oligo	3B, 3C, 8A
**17**	Yes	Yes	F	Yes	Extended Oligo	1A, 1B, 6C, 7C, 7D, 7E, 8A, 8B
**18**	Yes	Yes	F	Yes	Extended Oligo	8A, 8B, S3B
**19**	Yes	Yes	F	No	Persistent Oligo	8A, 8B
**20**	Yes	No	F	No	Persistent Oligo	8A, 8B
**21**	Yes	No	M	No	Extended Oligo	3B, 3C, 4A-C, 5A-H, 7D, 7E, 8A, 8B, S3A
**22**	Yes	No	M	No	Polyarticular	3A, 3C, 5D, 5F-H, 6A, 6B, 8A, 8B, S3A
**23**	Yes	No	F	No	Persistent Oligo	3B, 3C, 5D, 5F-H, 6A, 6B, 7D, 7E, 8A, 8B, S3A
**24**	Yes	No	M	Yes	Persistent Oligo	1C, 2A-D, 6C, 8A, 8B, S1A-C
**25**	Yes	No	F	No	Persistent Oligo	3A-C, 7E, 8A, 8B
**26**	Yes	No	M	No	Persistent Oligo	8A, 8B
**27**	Yes	No	F	No	Persistent Oligo	3B, 3C, 7D, 7E, 8A, 8B
**28**	Yes	No	M	No	Persistent Oligo	2B, 2D, 7E, 8A, 8B, S1A, S1C
**29**	Yes	No	F	No	Persistent Oligo	8A, 8B
**30**	Yes	No	M	No	Extended Oligo	3B, 3C, 7D, 7E, 8A, 8B
**31**	Yes	No	F	No	Persistent Oligo	3A-C, 5D, 5F-H, 6A, 6B, 8A, 8B, S3A
**32**	Yes	No	F	No	Persistent Oligo	8A, 8B, S3B
**33**	Yes	No	M	No	Persistent Oligo	3B, 3C, 7D, 7E, 8A, 8B
**34**	Yes	No	F	No	Persistent Oligo	3B, 3C, 7E, 8A, 8B
**35**	Yes	No	F	Yes	Polyarticular	3B, 3C, 5D, 5F-H, 6A, 6B, 7A, 7B, 7E, 8A, 8B, S3A
**36**	Yes	No	F	No	Polyarticular	3B, 3C, 7E, 8A, 8B
**37**	Yes	No	F	No	Polyarticular	8A, 8B
**38**	Yes	No	F	No	Polyarticular	6A, 6B, 8A, 8B
**39**	Yes	No	F	No	Polyarticular	8A, 8B, S3B
**40**	Yes	No	M	No	Polyarticular	3B, 3C, 5D, 5F-H, 7E, 8A, 8B, S3A
**41**	Yes	No	F	No	Polyarticular	3A, 3C, 8A, 8B

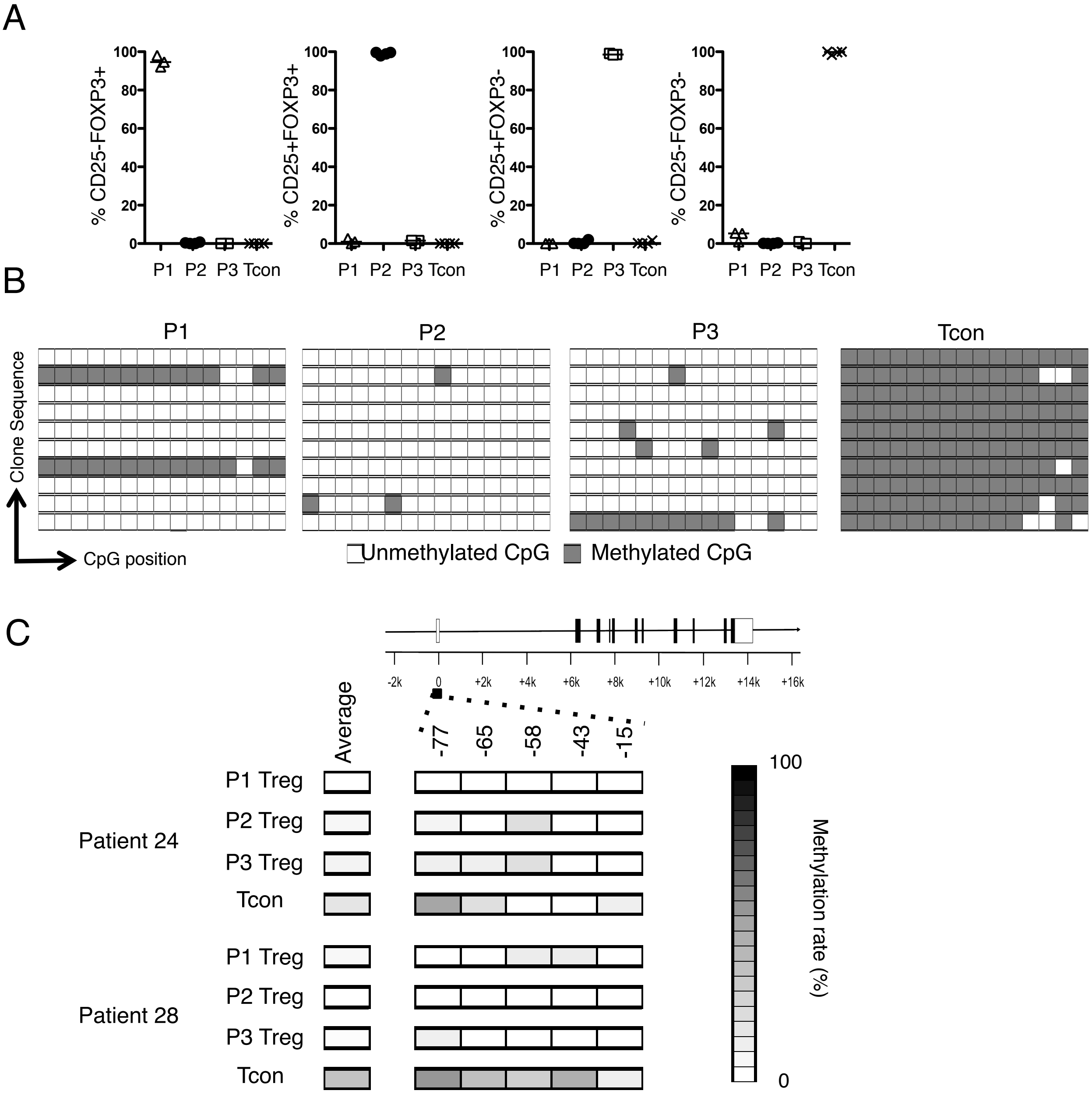

Supplementary Figure 1: TSDR sequence traces reveal small presence of non-Treg cells within P1 and P3 Treg populations, but complete demethylation at the FOXP3 promoter.

(A) Purity of sorted T cell populations isolated from the synovial fluid of patients 5, 9, 24 and 28 that were used for TSDR analysis in Fig. 2. (B) Displayed are representative TSDR sequences from 10 clones generated from the four T-cell populations in Fig. 2D from patient 24. Demethylated bases are shown in white, methylated CpGs are in grey tone. (C) FOXP3 promoter methylation status in the four T cell subsets from the synovial fluid of patients 24 and 28.

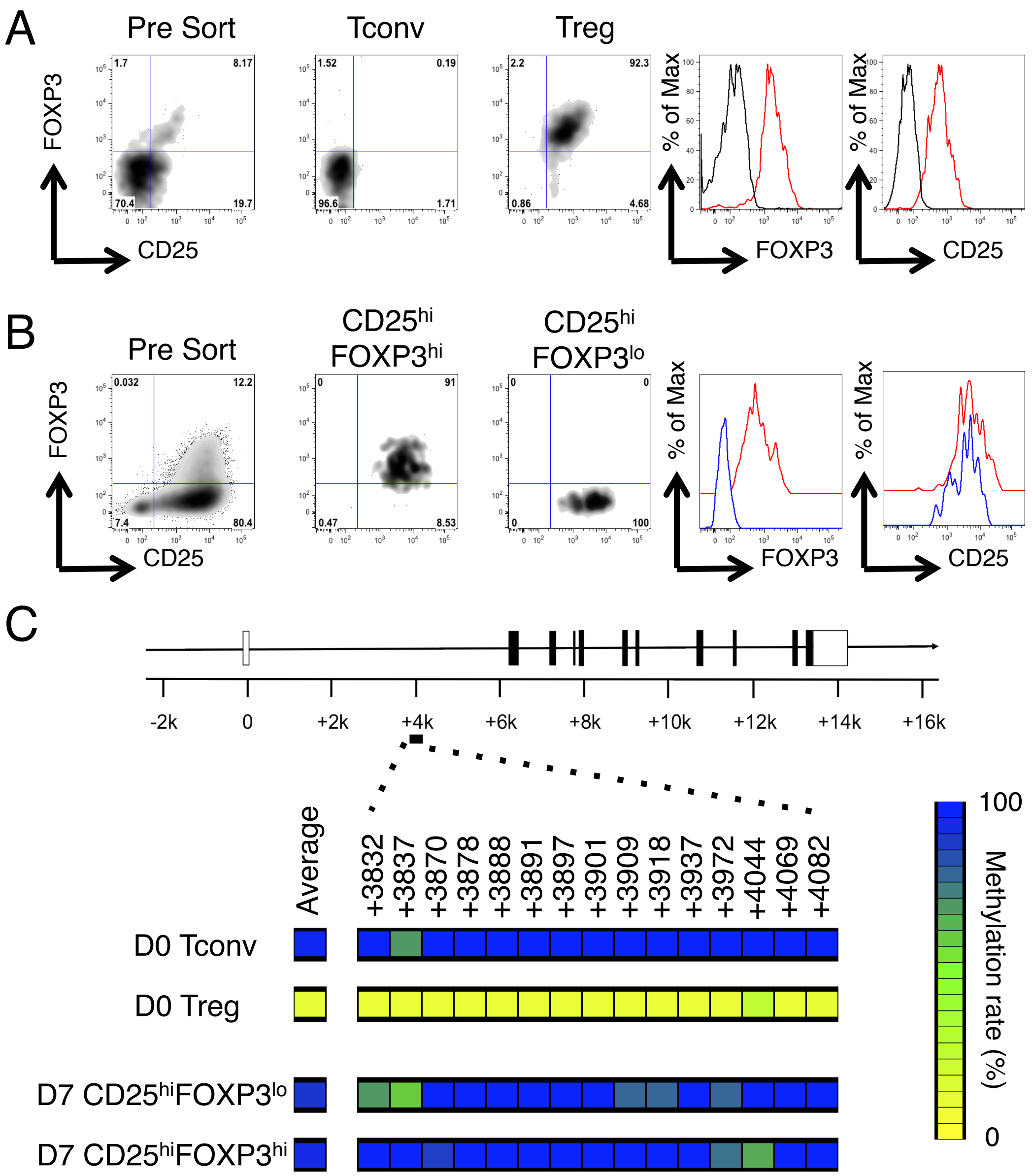

Supplementary Figure 2: In vitro-activated Tconv remain predominantly methylated at the TSDR irrespective of FOXP3 expression status.

CD4^{+} T-cells were isolated from healthy donor PBMC and stained for CD4, CD25 and CD127. Treg (CD25^{hi}CD127^{lo}) and Tconv (CD25^{lo}CD127^{hi}) were sorted and a proportion of cells taken for TSDR methylation analysis; remaining cells were cultured for 7 days on anti-CD3 and anti-CD28-coated plates in the presence of IL-2 before analysis of the TSDR region. (A) Unsorted CD4^{+} T-cells and day 0 sorted Treg and Tconv were stained for FOXP3. Flow cytometry plots display CD25 and FOXP3 expression in the three populations and histograms display the FOXP3 and CD25 expression levels (black=Tconv; red=Treg). (B) Following 7 days of culture, Tconv cells were harvested and stained for CD4, CD25 and FOXP3, before sorting in to CD25^{hi}FOXP3^{hi} and CD25^{hi}FOXP3^{lo} subsets. Flow cytometry plots displaying CD25 vs. FOXP3 expression, and histograms displaying FOXP3 or CD25 levels in the sorted subsets (CD25^{hi}FOXP3^{hi}=red; CD25^{hi}FOXP3^{lo}=blue). (C) DNA extracted from Treg and Tconv at day 0 and activated Tconv at day 7 was bisulfite treated, the TSDR region amplified, cloned and sequenced to determine the methylation status. Displayed are the heat maps of the methylation levels of 15 CpG positions, plus region average, in intron 1 of the *FOXP3* gene in the four populations. One of two independent experiments is shown.

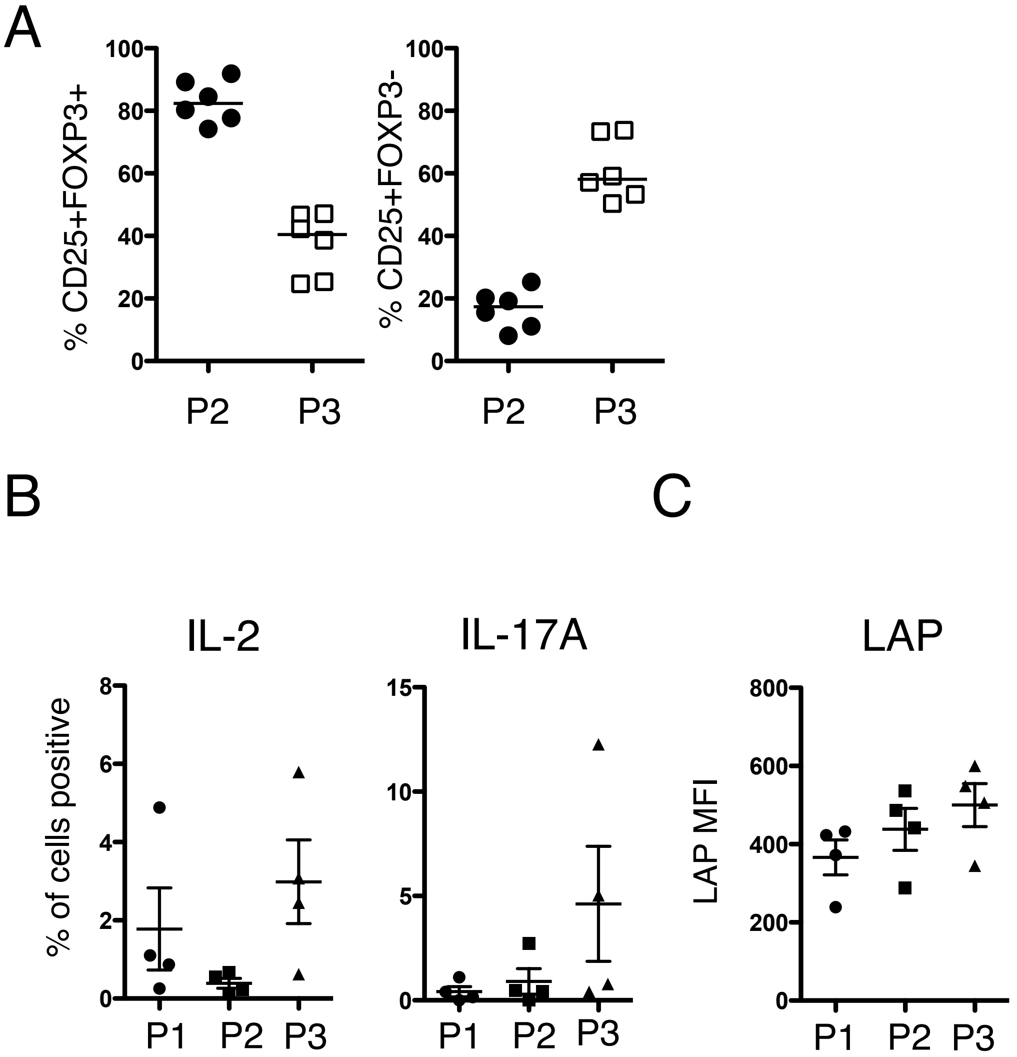

Supplementary Figure 3: P3 Treg show a small enrichment for IL-2 and IL-17A-producing cells but express comparable levels of the Latency-Associated Peptide (LAP).

(A) Purity of enriched P2 and P3 Treg populations used in Figure 5, based on CD25 and FOXP3 expression, n=6. (B) SFMC were stimulated for 2hrs with PMA and Ionomycin in the presence of Brefeldin A before staining for CD3, CD4, CD127, CD25, FOXP3, IL-2 and IL-17A. The frequency of IL-2 and IL-17A positive cells within the three Treg subsets was then determined, n=4 (C) SFMC were stained for CD3, CD4, CD127, CD25, FOXP3 and LAP and the expression of LAP quantified for each Treg subset. MFI = Median Fluorescence Intensity, n=4.
